# Functional Polymorphisms in the *TERT* Promoter Are Associated with Risk of Serous Epithelial Ovarian and Breast Cancers

**DOI:** 10.1371/journal.pone.0024987

**Published:** 2011-09-15

**Authors:** Jonathan Beesley, Hilda A. Pickett, Sharon E. Johnatty, Alison M. Dunning, Xiaoqing Chen, Jun Li, Kyriaki Michailidou, Yi Lu, David N. Rider, Rachel T. Palmieri, Michael D. Stutz, Diether Lambrechts, Evelyn Despierre, Sandrina Lambrechts, Ignace Vergote, Jenny Chang-Claude, Stefan Nickels, Alina Vrieling, Dieter Flesch-Janys, Shan Wang-Gohrke, Ursula Eilber, Natalia Bogdanova, Natalia Antonenkova, Ingo B. Runnebaum, Thilo Dörk, Marc T. Goodman, Galina Lurie, Lynne R. Wilkens, Rayna K. Matsuno, Lambertus A. Kiemeney, Katja K. H. Aben, Tamara Marees, Leon F. A. G. Massuger, Brooke L. Fridley, Robert A. Vierkant, Elisa V. Bandera, Sara H. Olson, Irene Orlow, Lorna Rodriguez-Rodriguez, Linda S. Cook, Nhu D. Le, Angela Brooks-Wilson, Linda E. Kelemen, Ian Campbell, Simon A. Gayther, Susan J. Ramus, Aleksandra Gentry-Maharaj, Usha Menon, Shahana Ahmed, Caroline Baynes, Paul D. Pharoah, kConFab Investigators, Australian Ovarian Cancer Study Group, ABCTB Investigators, Kenneth Muir, Artitaya Lophatananon, Arkom Chaiwerawattana, Surapon Wiangnon, Stuart Macgregor, Douglas F. Easton, Roger R. Reddel, Ellen L. Goode, Georgia Chenevix-Trench, on behalf of the Ovarian Cancer Association Consortium

**Affiliations:** 1 Division of Genetics and Population Health, Queensland Institute of Medical Research, Brisbane, Queensland, Australia; 2 Cancer Research Unit, Children's Medical Research Institute, Westmead, New South Wales, Australia; 3 Sydney Medical School, University of Sydney, Sydney, New South Wales, Australia; 4 Department of Oncology, University of Cambridge, Strangeways Research Laboratory, Cambridge, United Kingdom; 5 Department of Health Sciences Research, Mayo Clinic College of Medicine, Rochester, Minnesota, United States of America; 6 Department of Community and Family Medicine, Duke University Medical Center, Durham, North Carolina, United States of America; 7 Vesalius Research Center, VIB, Leuven, Belgium; 8 Vesalius Research Center, University of Leuven, Leuven, Belgium; 9 Division of Gynaecologic Oncology, Department of Obstetrics and Gynaecology, University Hospitals Leuven, University of Leuven, Leuven, Belgium; 10 Division of Cancer Epidemiology, German Cancer Research Center (DKFZ), Heidelberg, Germany; 11 Institute for Medical Biometrics and Epidemiology, University Clinic Hamburg-Eppendorf, Hamburg, Germany; 12 Department of Obstetrics and Gynecology, University of Ulm, Ulm, Germany; 13 Clinics of Obstetrics and Gynaecology, Hannover Medical School, Hannover, Germany; 14 Byelorussian Institute for Oncology and Medical Radiology Aleksandrov N.N., Minsk, Belarus; 15 Clinics of Radiation Oncology, Hannover Medical School, Hannover, Germany; 16 Clinics of Obstetrics and Gynaecology, Friedrich Schiller University, Jena, Germany; 17 Cancer Epidemiology Program, University of Hawaii Cancer Center, Honolulu, Hawaii, United States of America; 18 Department of Epidemiology, Biostatistics and HTA, Radboud University Nijmegen Medical Centre, Nijmegen, The Netherlands; 19 Department of Urology, Radboud University Nijmegen Medical Centre, Nijmegen, The Netherlands; 20 Comprehensive Cancer Center, The Netherlands, Nijmegen, The Netherlands; 21 Department of Gynaecology, Radboud University Nijmegen Medical Centre, Nijmegen, The Netherlands; 22 The Cancer Institute of New Jersey/Robert Wood Johnson Medical School, New Brunswick, New Jersey, United States of America; 23 Department of Epidemiology & Biostatistics, Memorial Sloan Kettering Cancer Center, New York, New York, United States of America; 24 Department of Population Health Research, Alberta Health Services-Cancer Care, Calgary, Alberta, Canada; 25 Peter MacCallum Cancer Centre, East Melbourne, Victoria, Australia; 26 Department of Preventive Medicine, Keck School of Medicine, University of Southern California, Los Angeles, California, United States of America; 27 Department of Gynaecological Oncology, University College London, EGA Institute for Women's Health, London, United Kingdom; 28 Department of Gynaecological Oncology and Westmead Institute for Cancer Research, University of Sydney at the Westmead Millennium Institute, Westmead Hospital, Sydney, New South Wales, Australia; 29 Westmead Millennium Institute, Sydney Medical School, Westmead, The University of Sydney, Sydney, New South Wales, Australia; 30 Health Sciences Research Institute, Warwick Medical School, University of Warwick, Coventry, United Kingdom; 31 Department of Academic Support, The National Cancer Institute of Thailand, Ministry of Public Health, Nonthaburi, Thailand; 32 Department of Pediatrics, Medical School, Khon Kaen University, Khon Kaen, Thailand; University of Pennsylvania School of Medicine, United States of America

## Abstract

Genetic variation at the *TERT-CLPTM1L* locus at 5p15.33 is associated with susceptibility to several cancers, including epithelial ovarian cancer (EOC). We have carried out fine-mapping of this region in EOC which implicates an association with a single nucleotide polymorphism (SNP) within the *TERT* promoter. We demonstrate that the minor alleles at rs2736109, and at an additional *TERT* promoter SNP, rs2736108, are associated with decreased breast cancer risk, and that the combination of both SNPs substantially reduces TERT promoter activity.

## Introduction

Genome-wide association studies (GWAS) have identified more than 140 cancer susceptibility loci for 17 different cancers (www.genome.gov/gwastudies), including a locus at 5p15.33 which has been implicated in susceptibility to melanoma [1], [2], glioma [3], lung [4], [5], pancreas [6], prostate [2], testicular [7], and bladder cancers [8]. This locus harbours *TERT*, encoding the reverse transcriptase component of telomerase, and cleft lip and palate transmembrane protein 1-like (*CLPTM1L*). GWAS of ovarian and breast cancer have not detected an association with this locus to date, which may be due to poor tagging of this region on the chips employed, or the lack of statistical power to detect associations. Using a candidate gene approach we previously reported evidence of an association between an intronic SNP in *TERT* (rs7726159) and EOC risk, particularly of the serous histological subtype, using a per-allele model [9]. In this study we carried out fine-mapping, followed by functional analyses of associated SNPs identified within the promoter region of *TERT*. We directly demonstrate that the presence of a common haplotype, which is associated with decreased cancer risk, substantially reduces TERT promoter activity. The vast majority of cancers depend on expression of telomerase, which requires substantial upregulation of TERT expression, for their continued proliferation, strongly implicating *TERT* as the predominant gene involved in this association.

## Results and Discussion

**Figure 1:**
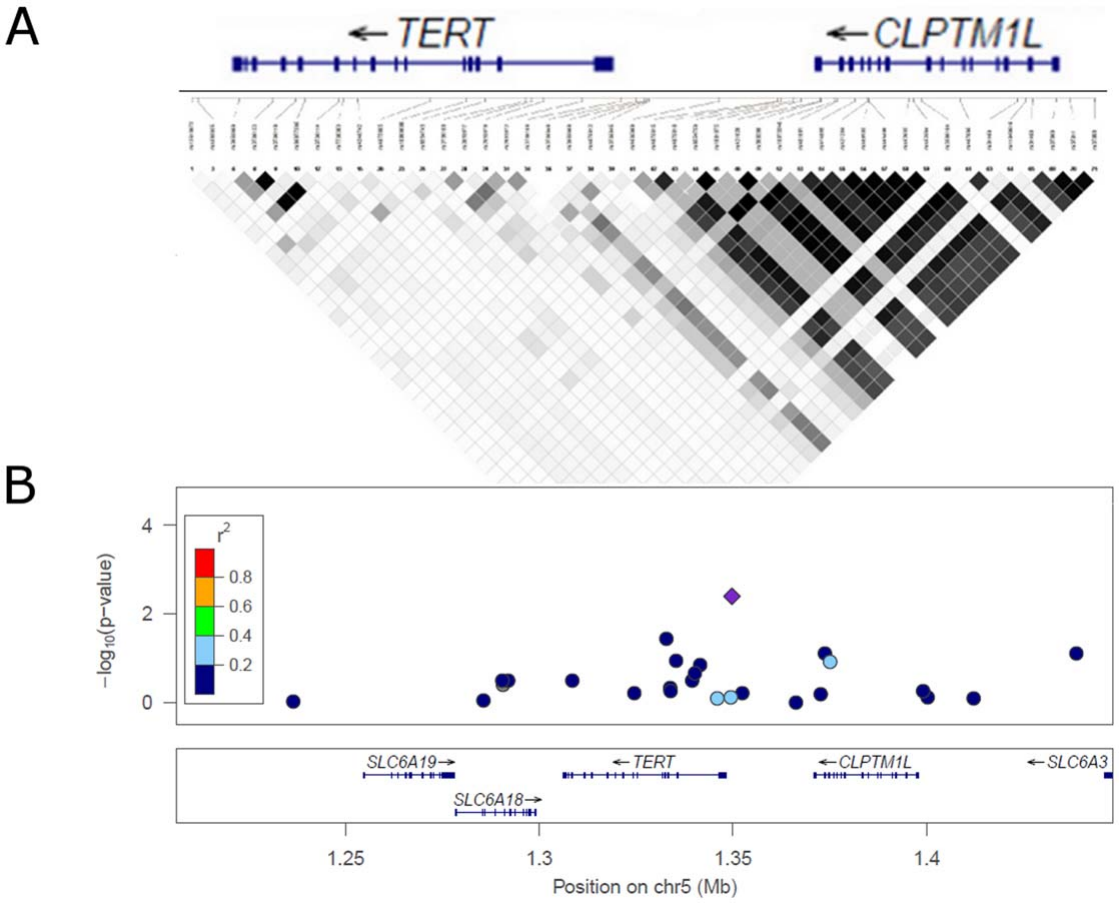
*TERT-CLPTM1L* locus SNPs genotyped in epithelial ovarian cancer cases and controls. Panel **A** depicts the pattern of linkage disequilibrium using data from the HapMap phase II+III CEU population (2010), where white represents r^2^ = 0, and black r^2^ = 1. The plot in **B** represents −log_10_ *p* against the chromosomal position for genotyped SNPs in the ovarian cancer fine mapping panel. The purple diamond represents the SNP (rs2736109) with the strongest observed association. The point colours represent the strength of LD according to 1000 Genomes Data.

To further clarify the association previously reported with *TERT*, we employed a fine-mapping strategy in nine case-control studies from the Ovarian Cancer Association Consortium (OCAC) (**Table S1**). SNPs in a region spanning ±250 kb across the *TERT*, *CLPT1ML*, *SLC6A18* and *SLC6A19* genes that were correlated (0.2≤r^2^≤0.99) with rs7726159, rs11133719, rs2735940 and rs2736100 [9] were selected from the 1000 Genomes low coverage pilot release of April 2009. The SNPs implicated by our previous study of EOC [9] (rs11133719, rs7726159, rs2736100 and rs2735940) or by cancer GWAS (rs2736100) were also included in the panel. We genotyped 36 SNPs by iPLEX (Sequenom Inc.) in 2,130 invasive EOC cases and 3,975 controls, all of Caucasian ancestry. After excluding monomorphic loci (n = 6) and two SNPs that failed OCAC's quality control criteria [10], 28 SNPs were analysed for association with risk of EOC. We used single marker and stepwise logistic regression models, adjusted for study and age (at interview for controls and at diagnosis for cases) with a threshold of *P*≤0.05 for addition (forward stepwise) or removal (backward stepwise) of SNPs (**Table S2**). We found that rs2736109 showed the strongest association with serous EOC (adjusted OR_per-allele_ 0.86 (0.77–0.96), *P* = 0.005) (**Table S3**), but not with invasive EOC risk overall (adjusted OR_per-allele_ 0.96 (0.89–1.05), *P* = 0.38) (data not shown). Likelihood ratio tests comparing logistic regression models with and without a genotype-by-study interaction term revealed no significant study heterogeneity (*P* = 0.4). rs2736109 is in a region of low linkage disequilibrium that encompasses the 5′ end of the *TERT* gene and the *TERT* promoter (**Figure 1**). This region also contains the SNPs, rs2736108 and rs2853669, which have pairwise correlations (r^2^) with each other, and with rs2736109 of greater than 0.6. It has previously been reported that rs2853669 is associated with breast cancer risk [11].

Since other loci confer susceptibility to both EOC and breast cancer [12], [13] we investigated associations of TERT SNPs with breast cancer risk in various data sets. First, we genotyped rs2736109 by iPLEX in 4,277 invasive breast cancer cases and 7,000 controls from Australian, German and Thai studies from the Breast Cancer Association Consortium (BCAC) (**Table S4**). In the combined analysis of the Australian and German studies, there was no association with invasive breast cancer risk overall (adjusted OR_per-allele_ 0.95 (0.90–1.01), *P* = 0.10), but there was an association in cases ≥50 years at diagnosis that approached significance (adjusted OR_per-allele_ 0.94 (0.88–1.00), *P* = 0.049). We also found a stronger association for ER-negative tumours (adjusted OR_per-allele_ 0.88 (0.80–0.98), *P* = 0.022; for ER-positive tumours adjusted OR_per-allele_ 0.98 (0.92–1.05), *P* = 0.562) (**Tables S5, S6, and S7**). We found the strongest evidence of association among ER-negative cases over the age of 50 (n = 636) (adjusted OR_per-allele_ 0.84 (0.75–0.95), *P* = 0.005). We did not find any association with breast cancer risk in a Thai study (n = 327 cases) genotyped for rs2736109.

Next, we analysed genotypes imputed using MaCH for rs2736109 (r^2^ = 0.45) from a breast cancer GWAS of 3,931 cases and 3,622 controls from the United Kingdom, from which neither age nor ER status were available [14]. We observed a significant association between rs2736109 and overall breast cancer risk in this population (OR_per-allele_ 0.91 (0.83–0.99), *P* = 0.037), and from a weighted meta-analysis of imputed and genotyped data from all studies (OR_per-allele_ 0.94 (0.89–0.98), *P* = 0.011).

In an additional replication sample set (SEARCH) we genotyped a correlated SNP, rs2736108 (r^2^ = 0.96, 1000 Genomes Project, Dec 2009, r^2^ = 0.63 based on 345 Australian controls) in 6,788 cases and 6,426 controls because rs2736109 was not amenable to genotyping by TaqMan (the genotyping platform used by this group). We observed a significant association with breast cancer risk overall (adjusted OR_per-allele_ 0.92 (0.87–0.97), *P* = 0.003), which was restricted to the subset of cases diagnosed at age 50 or older (adjusted OR_per-allele_ 0.91 (0.85–0.97), *P* = 0.002) (**Table S6**). Estimates according to ER status showed a significant association between the rs2736108 genotypes and ER-positive tumours (adjusted OR_per-allele_ 0.93 (0.88–0.99), *P* = 0.031); there was no significant association in ER-negative tumours but the estimated OR was similar (OR_per-allele_ 0.95 (0.85–1.06)) and the sample size much smaller. Comparison of models for both rs2736109 and rs2736108 with and without genotype by age group interaction terms showed no evidence of a statistical interaction on the multiplicative scale (*P*_interaction_≥0.25).

**Figure 2:**
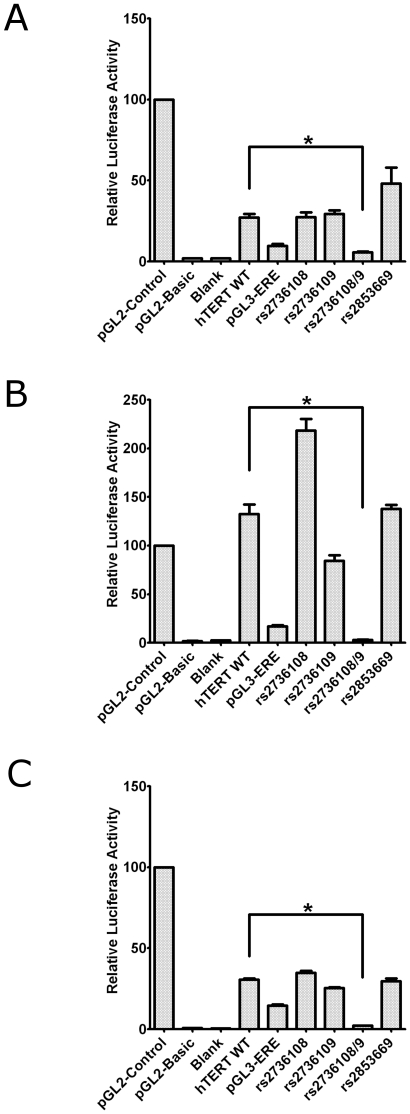
*TERT* promoter activity. Luciferase reporter assays following transient transfection with pGL2-control (SV40 promoter and enhancer), pGL2-basic (lacks promoter and enhancer), transfection control (blank), and the *TERT* reporter vectors *TERT* WT (3.9 kb of *TERT* promoter), the positive control *TERT*-ERE (containing a mutation in the estrogen-responsive element), and the minor alleles of rs2736108 (A allele), rs2736109 (A allele), rs2736108/9 (A alleles at both sites), rs2853669 (C allele) in (**A**) an EOC cell line (27/87), (**B**) a breast adenocarcinoma cell line (MDA-MB-468) and (**C**) a normal breast epithelial cell strain (Bre16). Error bars represent standard deviation between three separate experiments. * represents statistical significance using one-way ANOVA with post hoc Dunnett's tests.

The SNPs of interest (rs2736108, rs2736109 and rs2853669) lie within the upstream promoter region of *TERT*. To determine the functional significance of these sites, we generated combinations of these variants in a luciferase reporter construct containing 3.9 kb of the *TERT* promoter [15]. Relative promoter activity was determined in an EOC cell line (27/87), a breast adenocarcinoma cell line (MDA-MB-468), and in post-selection normal breast epithelial cells (Bre16) (**Figure 2**). Introduction of a mutation into the human estrogen-responsive element in the *TERT* promoter (TERT-ERE) [16] was used as a positive control and confirmed diminished reporter activity. In all three cell types, luciferase activity was substantially reduced for the construct carrying both minor (A) alleles at rs2736108 and rs2736109, but remained unaltered for those with the individual minor allele at either SNP. We observed no change in expression for the minor allele at rs2853669.

Our analysis of Australian controls estimated a frequency of 32% for the A-A haplotype at rs2736108 and rs2736109, suggesting that this relatively common promoter haplotype may lower the risk of ovarian and breast cancer through decreasing *TERT* expression. This finding provides no support for the hypothesis that decreased telomerase activity predisposes to genomic instability and consequent oncogenic progression but instead our data imply the opposite, namely, that *decreased* TERT expression confers *decreased* cancer risk.

We also examined tumour expression and germline variants at the *TERT-CLPTM1L* locus using data from The Cancer Genome Atlas (TCGA) ovarian serous cystadenocarcinoma set. mRNA expression profiling was available for 574 tumour samples using Affymetrix U133A platform This assay has one probe for *TERT*, but none for *CLPTM1L*. *TERT* was expressed at low levels, as expected, but this does not necessarily reflect low levels of telomerase protein expression or activity [17]. 508 normal DNA samples were genotyped using Illumina 1 M array, and the SNP rs2736108 was typed on this array. We also imputed the TCGA samples with reference to the 1000 Genome data (June 2010 release); rs2853669 was successfully imputed, but rs2736109 failed quality control. There was no evidence suggesting that the genotyped SNP, rs2736108, or the imputed SNP, rs2853669, are associated with expression of *TERT*. However one SNP, rs2735845, in partial linkage disequilibrium with the typed SNP, rs2736108 (r^2^ = 0.306), was associated with significantly altered transcript abundance of *TERT* (*P* = 4×10^−4^ and *P* = 0.0029 after correction for multiple testing). Gene copy number aberration was found in *TERT*; it was amplified in ∼20% of tumour samples. This SNP showed robust association after adjusting for the copy number variation (*P* = 1.8×10^−3^ and *P* = 0.015 after correction for multiple testing), and it explained 1.85% of variance of *TERT* transcription in ovarian cancer tumours.

A recently published meta-analysis identified a significantly decreased risk of breast cancer with a *TERT* SNP, rs2853669 (OR 0·76 (0·64–0·91), *P* = 0·002) [11]. This SNP lies in the same linkage disequilibrium block as rs2736108 and rs2736109 (r^2^>0.6 with rs2736108); therefore, this meta-analysis provides additional support for an association between *TERT* promoter SNPs and breast cancer risk. However, our functional analyses, which were carried out in normal breast epithelial cells and a breast adenocarcinoma cell line did not indicate any change to TERT expression with this individual SNP.

We computed a gene-based test [18] of association at *TERT* and *CLPTM1L* which yielded evidence in GWAS data available from dbGAP (http://www.ncbi.nlm.nih.gov/gap) for association with risk of lung, prostate and pancreatic cancer, but not breast cancer overall. Combining data for all cancers in a cross-cancer meta-analysis, revealed a genome-wide significant gene-based *P* = 4.1×10^−7^ for *CLPTM1L* (*P* = 0.008 after correction for 19,000 genes). A similar result was obtained for *TERT* (all cancer *P* = 7.7×10^−5^). This gene-based test includes all SNPs within 50 kb of the start/stop site of each gene. Most of the associated SNPs lie in the interval between *TERT* and *CLPTM1L* but with slightly more evidence for SNPs near to *CLPTM1L*, leading to a slightly higher gene-based *P* value for *CLPTM1L*. These results are based on marker data from Illumina GWAS arrays and hence the exact location of the maximum association test statistic is dependent to some degree upon the arbitrary set of SNPs that are on the arrays. Clearly, additional analysis of the entire *TERT-CLPM1L* locus is warranted in breast, and other, cancers to validate the associations we have identified and fine map putative causal variants if our findings are confirmed.

The failure to date to identify an association between the *TERT-CLPTM1L* locus and risk of EOC or breast cancer by GWAS may be explained by the pattern of linkage disequilibrium of the relevant SNPs: rs2736100, the tagSNP most commonly identified by GWAS of other cancers is poorly correlated with rs2736108 and rs2736109 (r^2^ = 0.141 and 0.105, respectively), and neither of these SNPs are on the Illumina 300 K, 610 K or 650 K arrays used for most cancer GWAS.

In summary, we have demonstrated a direct association between functional SNPs in the *TERT* promoter, which confer decreased risk of ovarian and breast cancer, and reduced TERT promoter activity. Decreased levels of TERT result in progressive telomere shortening and the onset of cellular senescence, which ultimately acts to suppress tumorigenesis. The association of hypomorphic sequence variants in the *TERT* promoter with decreased risk of cancer implicates downregulation of telomerase and telomere shortening as an intrinsic tumour suppressive mechanism. It is also possible that TERT variants associated with elevated cancer risk may alter the stringency with which TERT is regulated, potentially facilitating TERT activation and consequently providing a tumorigenic advantage. Potential non-canonical roles of TERT in cell signalling pathways may also underlie cancer risk [19]. Our results add functional insight into the increasingly important role of *TERT* as a cancer risk factor and demonstrate the need for further mechanistic analysis of this multi-cancer susceptibility locus.

## Materials and Methods

### Ethics Statement

Approval for this study was obtained from The Queensland Institute of Medical Research Human Research Ethics Committee. All studies were approved by the review boards and ethics committees of their respective institutions, and all participants provided written informed consent.

### Genotyping

iPLEX genotyping was carried out using MALDI-TOF spectroscopy utilising Sequenom's MassARRAY platform and iPLEX GOLD chemistry. 10 ng of genomic DNA was used as template, to which a PCR mix containing Qiagen Hot-StarTaq was added. Shrimp alkaline phosphatase and primer extension steps were carried out using Sequenom's protocol and reagents. Primers were obtained from Integrated DNA Technologies (Ohio USA). Assays were designed with MassARRAY Assay Design version 3.1(Sequenom). Raw genotype data were visualised and processed with MassARRAY Typer software version 3.4. TaqMan genotyping (SEARCH) was carried out as previously described [20]. Strict quality control criteria were adhered to as part of the Ovarian Cancer Association Consortium's guidelines [10].

### Statistical methods

We used single marker and stepwise logistic regression models to screen 28 SNPs in non-Hispanic white ovarian cancer cases (n = 2,130) and controls (n = 3,975) from nine OCAC studies (Supplementary Table 1). Genotype data for all the previously reported *TERT* SNPs [9] has been excluded from the current analysis. Stepwise models were adjusted for study and age (at interview for controls and at diagnosis for cases), with a threshold of *P*≤0.05 for addition (forward stepwise) or removal (backward stepwise) of SNPs. All single marker risk estimates were obtained from unconditional logistic regression models adjusted for age (where available) and additionally for study where data was pooled across multiple studies. Assuming a log additive model of inheritance, the per-allele odds ratios (ORs) and their 95% confidence intervals (CIs) associated for selected SNPs were estimated by fitting the number of rare alleles carried as a continuous covariate. Ovarian cancer risk associated with SNP genotypes were obtained for all invasive cases as well as a subset of serous cases. Breast cancer risk estimates were obtained for invasive cases and by estrogen receptor (ER) status. Separate comparisons were made for cases diagnosed before 50 vs. ≥50 years of age to explore effect modification by advancing age of diagnosis. Summary estimates from pooled analyses using genotyped and imputed SNP data were obtained from weighted meta-analysis of study-specific parameter estimates (β coefficients and Standard Error). The minor allele frequency (MAF) for each SNP was estimated from the control population for each study. Study heterogeneity and risk differences associated with age groups (<50 vs. ≥50) were assessed using the likelihood ratio test to compare logistic regression models with and without a multiplicative interaction term. All tests for association were two-tailed, statistical significance was assessed at *P*≤0.05, and were performed in STATA SE v.11 (StataCorp, USA), and SAS v. 9.1. Tests for study heterogeneity and age group interaction tests were implemented in the R project for Statistical Computing (http://www.r-project.org/).

### VEGAS applied to dbGAP data

To evaluate evidence for association at *TERT* and *CLPTM1L* with various cancers, we applied the gene-based test implemented in VEGAS (all SNPs in gene test) [18] to data from dbGAP. In brief, we selected cancer cases and controls from dbGAP that were genotyped on ∼550,000 SNPs (Illumina 610 quad or Illumina HumanHap550 arrays). Studies were CGEMS breast (1145 cases, 1142 controls), CGEMS pancreatic cancer (2328 cases, 2351 controls), GENEVA lung cancer (EAGLE and PLCO combined 2748 cases, 2840 controls) and CGEMS prostate (1145 cases, 1054 controls). For full details see dbGAP website (http://www.ncbi.nlm.nih.gov/gap). In each case, following standard quality control, the genomic control lambda was as would be expected if cases and controls were well matched; breast, pancreatic, lung, prostate lambda values 1.01, 1.01, 1.03, 1.03, respectively. The VEGAS gene based *P* values from each of the four dbGAP studies were combined in a meta-analysis using Fisher's method for combining *P* values.

### Luciferase assays

Variants were introduced into pGL3-hTERT-3915 [15] by site-directed mutagenesis (Agilent Technologies). The A-A vector was generated by introducing the variants sequentially. Cells were transfected using siPORT *NeoFX* Transfection Agent (Ambion), according to the manufacturer's instructions, and harvested after 48 h. Cells were washed with phosphate buffered saline (PBS) and lysed with 200 µL/well lysis buffer (25 mM tris pH 7.8, 2 mM EDTA, 10% glycerol, 1% Triton X-100, 0.2 mM DTT). Luciferase activity was assayed in triplicate for each transfection using 20 µL lysate and 50 µL reconstituted luciferase assay reagent (Promega). Luminescence was measured immediately for 4 s in each well with a Wallac Victor^3^ 1420 multilabel counter. The experiment was repeated three times and the results averaged. Data were analysed by one-way ANOVA with post hoc Dunnett's tests in GraphPad Prism version 5.03 (GraphPad Software).

## Supporting Information

Table S1 Participating EOC case-control studies
(DOC)

Table S2 Per allele OR for all SNPs in EOC
(DOC)

Table S3 The association of rs2736109 with EOC, by study
(DOC)

Table S4 Participating invasive breast case-control studies
(DOC)

Table S5 The association of rs2736109/rs2736108 with risk of invasive breast cancer, by study
(DOC)

Table S6 Breast cancer risk by age
(DOC)

Table S7 Breast cancer risk by ER status
(DOC)
