# Supplementary material for: Functional Polymorphisms in the TERT Promoter Are Associated with Risk of Serous Epithelial Ovarian and Breast Cancers
Source: PLoS One. 2011 Sep 15;6(9):e24987. doi: 10.1371/journal.pone.0024987 (PMC3174246; doi:10.1371/journal.pone.0024987)
Supplement: Table S1 — Participating EOC case-control studies (DOC) [file pone.0024987.s001.doc]

**Table S1:** Distribution of Caucasian cases and controls from participating Ovarian Cancer Association Consortium case-control studies

| **Study** | **OCAC Acronym** | **Country** | **Invasive (serous) EOC cases** | **Controls** |
| --- | --- | --- | --- | --- |
| Belgium Ovarian Cancer Study | BEL | Belgium | 189 (123) | 434 |
| Germany Ovarian Cancer Study | GER | Germany | 225 (107) | 429 |
| Hawaii Ovarian Cancer Study | HAW | USA | 84 (45) | 166 |
| Hannover-Jena Ovarian Cancer Study/ Hannover-Minsk Ovarian Cancer Study | HJO/HMO | Germany | 246 (71) | 897 |
| Mayo Clinic Ovarian Cancer Case Control Study | MAY | USA | 399 (254) | 477 |
| New Jersey Ovarian Cancer Study | NJO | USA | 173 (103) | 179 |
| Nijmegen Polygene Study & Nijmegen Biomedical Study | NTH | Netherlands | 293 (101) | 586 |
| Ovarian Cancer in Alberta and British Columbia Study | OVA | Canada | 452 (236) | 420 |
| Southampton Ovarian Cancer Study/ UK Ovarian Cancer Population Study | SOC/UKO | UK | 69 (36) | 387 |
| **Totals** |  |  | **2130 (1076)** | **3975** |
