# Supplementary material for: Functional Polymorphisms in the TERT Promoter Are Associated with Risk of Serous Epithelial Ovarian and Breast Cancers
Source: PLoS One. 2011 Sep 15;6(9):e24987. doi: 10.1371/journal.pone.0024987 (PMC3174246; doi:10.1371/journal.pone.0024987)
Supplement: Table S2 — Per allele OR for all SNPs in EOC (DOC) [file pone.0024987.s002.doc]

**Table S2** Per allele OR for association of *TERT* region SNPs with serous epithelial ovarian cancer

| **SNP** | **Common/rare allele** | **Gene** | **Location** | **MAF** | **Cases** | **Controls** | **OR (95% CI)** | ***P*** |
| --- | --- | --- | --- | --- | --- | --- | --- | --- |
| rs11750211 | C/T |  |  | 0.26 | 801 | 3250 | 0.97 (0.85-1.11) | 0.684 |
| rs4975622 | G/T | *SLC6A18* | Intron | 0.31 | 799 | 3252 | 1.00 (0.89-1.14) | 0.937 |
| rs7731934 | A/T | *SLC6A18* | Intron | 0.30 | 989 | 3693 | 1.05 (0.93-1.17) | 0.438 |
| rs60871464 | G/A | *SLC6A18* | Intron | 0.31 | 799 | 3252 | 1.05 (0.94-1.18) | 0.459 |
| rs7718658 | C/T | *SLC6A18* | Intron | 0.31 | 988 | 3690 | 1.05 (0.94-1.18) | 0.373 |
| rs33954691 | C/T | *TERT* | Coding exon | 0.11 | 990 | 3694 | 0.85 (0.72-1.02) | 0.077 |
| rs11133719 | G/A | *TERT* | Intron | 0.16 | 876 | 3610 | 0.95 (0.82-1.10) | 0.491 |
| rs10069690 | G/A | *TERT* | Intron | 0.25 | 989 | 3690 | 1.11 (0.99-1.25) | 0.076 |
| rs4975538 | G/C | *TERT* | Intron | 0.35 | 988 | 3687 | 1.08 (0.97-1.20) | 0.181 |
| rs6897196 | T/C | *TERT* | Intron | 0.39 | 990 | 3686 | 1.07 (0.96-1.19) | 0.211 |
| rs7726159 | G/T | *TERT* | Intron | 0.34 | 885 | 3373 | 1.08(0.96-1.22) | 0.170 |
| rs2736100 | T/G | *TERT* | Intron | 0.50 | 989 | 3689 | 0.98 (0.88-1.09) | 0.667 |
| rs2853677 | T/C | *TERT* | Intron | 0.42 | 989 | 3692 | 1.05 (0.94-1.17) | 0.393 |
| rs2853676 | G/A | *TERT* | Intron | 0.26 | 990 | 3687 | 1.05 (0.93-1.18) | 0.453 |
| k5_1345643 | T/A | *TERT* | Intron | 0.0004 | 799 | 3244 | 2.01 (0.20-20.45) | 0.555 |
| rs2853672 | G/T | *TERT* | Intron | 0.48 | 801 | 3248 | 1.04 (0.93-1.16) | 0.529 |
| rs2735940 | T/C | *TERT* | Promoter | 0.49 | 989 | 3692 | 1.00 (0.90-1.11) | 0.949 |
| **rs2736109** | **G/A** | ***TERT*** | **Promoter** | **0.41** | **969** | **3630** | **0.86 (0.77-0.96)** | **0.005** |
| rs2735846 | G/C | *TERT* | Promoter | 0.50 | 800 | 3250 | 0.95 (0.85-1.07) | 0.397 |
| rs36115365 | G/C | *CLPTM1L* | 3’ | 0.20 | 801 | 3244 | 1.00 (0.86-1.15) | 0.983 |
| rs451360 | G/T | *CLPTM1L* | Intron | 0.22 | 990 | 3683 | 1.03 (0.91-1.17) | 0.655 |
| rs402710 | C/T | *CLPTM1L* | Intron (boundary) | 0.34 | 783 | 3198 | 1.13 (1.00-1.27) | 0.055 |
| rs401681 | C/T | *CLPTM1L* | Intron | 0.43 | 801 | 3247 | 1.11 (0.99-1.25) | 0.076 |
| rs27071 | A/G | *CLPTM1L* | Promoter | 0.25 | 798 | 3251 | 1.03 (0.90-1.17) | 0.708 |
| rs27068 | G/A | *CLPTM1L* | Promoter | 0.25 | 991 | 3688 | 1.00 (0.89-1.13) | 0.938 |
| rs6890396 | C/T |  |  | 0.26 | 990 | 3691 | 1.07 (0.95-1.20) | 0.297 |
| rs11744775 | G/A | *SLC6A3* | 3’ | 0.23 | 801 | 3250 | 1.10 (0.96-1.26) | 0.158 |
| rs4975553 | T/A |  |  | 0.13 | 797 | 3253 | 0.86 (0.72-1.03) | 0.103 |
