# Supplementary material for: Functional Polymorphisms in the TERT Promoter Are Associated with Risk of Serous Epithelial Ovarian and Breast Cancers
Source: PLoS One. 2011 Sep 15;6(9):e24987. doi: 10.1371/journal.pone.0024987 (PMC3174246; doi:10.1371/journal.pone.0024987)
Supplement: Table S3 — The association of rs2736109 with EOC, by study (DOC) [file pone.0024987.s003.doc]

**Table S3** Per allele odds ratios for rs2736109 in serous, invasive EOC by site

| **Study** | **Cases** | **Controls** | **MAF** | **Adj OR (95% CI) *** | ***P*** |
| --- | --- | --- | --- | --- | --- |
| BEL | 106 | 421 | 0.39 | 1.02 (0.69-1.52) | 0.92 |
| GER | 102 | 417 | 0.42 | 0.73 (0.53-1.01) | 0.05 |
| HJO/HMO | 69 | 749 | 0.43 | 0.89 (0.55-1.44) | 0.64 |
| HAW | 43 | 154 | 0.44 | 0.70 (0.42-1.16) | 0.17 |
| MAY | 249 | 469 | 0.40 | 0.99 (0.79-1.23) | 0.92 |
| NJO | 98 | 175 | 0.42 | 0.92 (0.62-1.37) | 0.68 |
| NTH | 53 | 484 | 0.41 | 0.67 (0.43-1.02) | 0.06 |
| OVA | 215 | 382 | 0.42 | 0.73 (0.57-0.93) | 0.01 |
| SOC/UKO | 34 | 382 | 0.42 | 0.94 (0.55-1.61) | 0.83 |
| **Total** | **969** | **3633** | **0.41** | **0.86 (0.77-0.96)** | **0.005** |

*Adjusted for age
