# Supplementary material for: Functional Polymorphisms in the TERT Promoter Are Associated with Risk of Serous Epithelial Ovarian and Breast Cancers
Source: PLoS One. 2011 Sep 15;6(9):e24987. doi: 10.1371/journal.pone.0024987 (PMC3174246; doi:10.1371/journal.pone.0024987)
Supplement: Table S4 — Participating invasive breast case-control studies (DOC) [file pone.0024987.s004.doc]

**Table S4** Breast Cancer Association Consortium studies used in analysis of *TERT* promoter SNP associations

| **Study** | **BCAC Acronym** | **Country** | **Study Type** | **Cases** | **Controls** |
| --- | --- | --- | --- | --- | --- |
| Australian Breast Cancer Tissue Bank | ABCTB | Australia | Case-only | 671 | - |
| Kathleen Cuningham Consortium for Research into Familial Breast Cancer | kConFab | Australia | Familial cases only | 447 | - |
| Australian Ovarian Cancer Study | AOCS | Australia | Case-control | - | 934 |
| Genetic Epidemiology Study of Breast Cancer by Age 50 | GESBC | Germany | Case-control | 445 | 554 |
| Mammary Carcinoma Risk Factor Investigation | MARIE | Germany | Case-control | 2387 | 4945 |
| Study of Epidemiology and Risk factors in Cancer Heredity | SEARCH | UK | Case-control | 6788 | 6426 |
| Asia Cancer Program | ACP | Thailand | Case-control | 327 | 567 |

All the studies were genotyped by iPLEX for rs2736109, except SEARCH which was genotyped by TaqMan for rs2736108

* Cases from ABCTB and kConFab were compared to controls from AOCS for all analyses
