# Supplementary material for: Functional Polymorphisms in the TERT Promoter Are Associated with Risk of Serous Epithelial Ovarian and Breast Cancers
Source: PLoS One. 2011 Sep 15;6(9):e24987. doi: 10.1371/journal.pone.0024987 (PMC3174246; doi:10.1371/journal.pone.0024987)
Supplement: Table S5 — The association of rs2736109/rs2736108 with risk of invasive breast cancer, by study (DOC) [file pone.0024987.s005.doc]

**Table S5** Association of SNPs in the *TERT* promoter and **overall** breast cancer risk

| **Study** | **SNP** | **Genotype** | **Controls (%)** | **Cases (%)** | **MAF ^a^** | **OR (95% CI)** | ***P*** |
| --- | --- | --- | --- | --- | --- | --- | --- |
| **AUS ^b^** | rs2736109 | GG | 335 (35.87) | 402 (35.96) | 0.41 | 1.00 |  |
|  |  | GA | 435 (46.57) | 547 (48.93) |  | 1.05 (0.86-1.27) |  |
|  |  | AA | 164 (17.56) | 169 (15.12) |  | 0.86 (0.66-1.11) |  |
|  |  | per A allele |  |  |  | 0.95 (0.84-1.07) | 0.411 |
| **GESBC** | rs2736109 | GG | 189 (34.12) | 162 (36.40) | 0.41 | 1.00 |  |
|  |  | GA | 278 (50.18) | 212 (47.64) |  | 0.89 (0.67-1.17) |  |
|  |  | AA | 87 (15.70) | 71 (15.96) |  | 0.95 (0.65-1.39) |  |
|  |  | per A allele |  |  |  | 0.96 (0.80-1.15) | 0.642 |
| **MARIE** | rs2736109 | GG | 1645 (33.27) | 844 (35.36) | 0.42 | 1.00 |  |
|  |  | GA | 2443 (49.40) | 1136 (47.59) |  | 0.91 (0.81-1.01) |  |
|  |  | AA | 857 (17.33) | 407 (17.05) |  | 0.91 (0.80-1.07) |  |
|  |  | per A allele |  |  |  | 0.95 (0.89-1.02) | 0.171 |
| **COMBINED ^c^** | rs2736109 | GG | 2169 (33.72) | 1408 (35.65) | 0.42 | 1.00 |  |
|  |  | GA | 3156 (49.06) | 1895 (47.97) |  | 0.93 (0.85-1.02) |  |
|  |  | AA | 1108 (17.22) | 647 (16.38) |  | 0.91 (0.81-1.03) |  |
|  |  | per A allele |  |  |  | 0.95 (0.90-1.01) | 0.096 |
|  |  |  |  |  |  |  |  |
| **UK GWAS ^d^** | rs2736109 | per A allele | 3622 | 3931 |  | 0.91 (0.83 - 0.99) | **0.037** |
|  |  |  |  |  |  |  |  |
| **Meta-analysis^e^** | rs2736109 | Per A allele | 10055 | 7881 |  | 0.94 (0.89-0.98) | **0.011** |
|  |  |  |  |  |  |  |  |
| **SEARCH** | rs2736108 | GG | 3141 (48.88) | 3467 (51.08) | 0.30 | 1.00 |  |
|  |  | GA | 2710 (42.17) | 2790 (41.10) |  | 0.93 (0.87-1.00) |  |
|  |  | AA | 575 (8.95) | 531 (7.82) |  | 0.84 (0.74-0.95) |  |
|  |  | per A allele |  |  |  | 0.92 (0.87-0.97) | **0.003** |
|  |  |  |  |  |  |  |  |
| **ACP** | rs2736109 | GG | 202 (35.63) | 125 (38.23) | 0.40 | 1.00 |  |
|  |  | GA | 282 (49.74) | 142 (43.43) |  | 0.81 (0.60-1.10) |  |
|  |  | AA | 83 (14.64) | 60 (18.35) |  | 1.17 (0.78-1.74) |  |
|  |  | per A allele |  |  |  | 1.02 (0.84-1.24) | 0.818 |
| ^a^ Minor allele frequency in controls. ^b^ ABCTB and kConFab cases compared to AOCS controls. ^c^ adjusted for study. ^d^ Imputed genotypes. **^e^** Meta-analysis of the combined and UK-GWAS results | | | | | | | |
