# Supplementary material for: Functional Polymorphisms in the TERT Promoter Are Associated with Risk of Serous Epithelial Ovarian and Breast Cancers
Source: PLoS One. 2011 Sep 15;6(9):e24987. doi: 10.1371/journal.pone.0024987 (PMC3174246; doi:10.1371/journal.pone.0024987)
Supplement: Table S6 — Breast cancer risk by age (DOC) [file pone.0024987.s006.doc]

**Table S6** Association of *TERT* promoter SNPs and breast cancer risk in Caucasians by age

|  |  | **INVASIVE Cases, age < 50** | | | | **INVASIVE Cases, age ≥ 50** | | | |
| --- | --- | --- | --- | --- | --- | --- | --- | --- | --- |
| **Study** | **Genotype** | **Controls** | **Cases** | **OR (95% CI)** | ***P*** | **Controls** | **Cases** | **OR (95% CI)** | ***P*** |
| **AUS ^a^** | GG | 73 (38.02) | 127 (33.25) | 1.00 |  | 262 (35.31) | 275 (37.36) | 1.00 |  |
|  | GA | 89 (46.35) | 185 (48.43) | 1.20 (0.82-1.75) |  | 346 (46.63) | 362 (49.18) | 1.00 (0.80-1.25) |  |
|  | AA | 30 (15.63) | 70 (18.32) | 1.34 (0.80-2.24) |  | 134 (18.06) | 99 (13.45) | 0.70 (0.52-0.96) |  |
|  | per A allele | |  | 1.17 (0.91-1.49) | 0.229 |  |  | 0.87 (0.75-1.01) | 0.065 |
| **GESBC** | GG | 170 (33.46) | 152 (36.45) | 1.00 |  | 19 (41.30) | 10 (35.71) | 1.00 |  |
|  | GA | 259 (50.98) | 200 (47.96) | 0.86 (0.65-1.15) |  | 19 (41.30) | 12 (42.86) | 1.20 (0.42-3.44) |  |
|  | AA | 79 (15.55) | 65 (15.59) | 0.92 (0.62-1.37) |  | 8 (17.39) | 6 (21.43) | 1.42 (0.39-5.26) |  |
|  | per A allele | |  | 0.94 (0.78-1.14) | 0.514 |  |  | 1.19 (0.63-2.26) | 0.585 |
| **MARIE** | GG | 0 | 0 |  |  | 1645 (33.27) | 844 (35.36) | 1.00 |  |
|  | GA | 0 | 0 |  |  | 2443 (49.40) | 1136 (47.59) | 0.91 (0.81-1.01) |  |
|  | AA | 0 | 0 |  |  | 857 (17.33) | 407 (17.05) | 0.93 (0.80-1.07) |  |
|  | per A allele | |  |  |  |  |  | 0.95 (0.89-1.02) | 0.171 |
| **COMBINED ^b^** | GG | 243 (34.71) | 279 (34.92) | 1.00 |  | 1926 (33.59) | 1129 (35.83) | 1.00 |  |
| rs2736109 | GA | 348 (49.71) | 385 (48.19) | 0.97 (0.77-1.22) |  | 2808 (48.98) | 1510 (47.92) | 0.92 (0.84-1.02) |  |
|  | AA | 109 (15.57) | 135 (16.90) | 1.06 (0.77-1.44) |  | 999 (17.43) | 512 (16.25) | 0.89 (0.78-1.01) |  |
|  | per A allele | |  | 1.02 (0.88-1.18) | 0.830 |  |  | 0.94 (0.88-1.00) | **0.049** |
| **SEARCH** | GG | 673 (49.93) | 1103 (50.46) | 1.00 |  | 2468 (48.60) | 2364 (51.36) | 1.00 |  |
| rs2736108 | GA | 570 (42.28) | 921 (42.13) | 0.99 (0.86-1.14) |  | 2140 (42.14) | 1869 (40.61) | 0.91 (0.84-0.99) |  |
|  | AA | 105 (7.79) | 162 (7.41) | 0.94 (0.72-1.23) |  | 470 (9.26) | 369 (8.02) | 0.82 (0.71-0.95) |  |
|  | per A allele | |  | 0.98 (0.88-1.09) | 0.676 |  |  | 0.91 (0.85-0.97) | **0.002** |
| ^a^ ABCTB and kConFab cases compared to AOCS controls. ^b^ Adjusted for study in combined analysis. | | | | | | | | | |
