# Supplementary material for: Functional Polymorphisms in the TERT Promoter Are Associated with Risk of Serous Epithelial Ovarian and Breast Cancers
Source: PLoS One. 2011 Sep 15;6(9):e24987. doi: 10.1371/journal.pone.0024987 (PMC3174246; doi:10.1371/journal.pone.0024987)
Supplement: Table S7 — Breast cancer risk by ER status (DOC) [file pone.0024987.s007.doc]

**Table S7** Association of *TERT* promoter SNPs and breast cancer risk in Caucasians, by ER status

|  |  | **ER positive** | |  |  | **ER negative** | |  |  |
| --- | --- | --- | --- | --- | --- | --- | --- | --- | --- |
| **Study** | **Genotype** | **Controls (%)** | **Cases (%)** | **OR (95% CI)** | ***P*** | **Controls (%)** | **Cases (%)** | **OR (0.95-CI)** | ***P*** |
| **AUS ^a^** | GG | 335 (35.87) | 243 (35.06) | 1.00 |  | 335 (35.87) | 84 (39.07) | 1.000 |  |
|  | GA | 435 (46.57) | 342 (49.35) | 1.08 (0.87-1.35) |  | 435 (46.57) | 96 (44.65) | 0.88 (0.64-1.22) |  |
|  | AA | 164 (17.56) | 108 (15.58) | 0.91 (0.68-1.22) |  | 164 (17.56) | 35 (16.28) | 0.85 (0.55-1.32) |  |
|  | per A allele | |  | 0.98 (0.85-1.12) | 0.738 |  |  | 0.91 (0.74-1.13) | 0.403 |
| **GESBC** | GG | 189 (34.12) | 79 (37.09) | 1.00 |  | 189 (34.12) | 44 (34.38) | 1.00 |  |
|  | GA | 278 (50.18) | 97 (45.54) | 0.84 (0.59-1.18) |  | 278 (50.18) | 61 (47.66) | 0.94 (0.61-1.45) |  |
|  | AA | 87 (15.70) | 37 (17.37) | 1.02 (0.64-1.62) |  | 87 (15.70) | 23 (17.97) | 1.14 (0.65-2.00) |  |
|  | per A allele | |  | 0.97 (0.77-1.22) | 0.814 |  |  | 1.04 (0.79-1.38) | 0.766 |
| **MARIE** | GG | 1645 (33.27) | 641 (34.72) | 1.00 |  | 1645 (33.27) | 192 (37.94) | 1.00 |  |
|  | GA | 2443 (49.40) | 874 (47.35) | 0.92 (0.82-1.04) |  | 2443 (49.40) | 244 (48.22) | 0.86 (0.70-1.04) |  |
|  | AA | 857 (17.33) | 331 (17.93) | 0.99 (0.85-1.16) |  | 857 (17.33) | 70 (13.83) | 0.70 (0.53-0.93) |  |
|  | per A allele | |  | 0.98 (0.91-1.06) | 0.652 |  |  | 0.84 (0.74-0.96) | **0.012** |
| **COMBINED ^b^** | GG | 2169 (33.72) | 963 (34.99) | 1.00 |  | 2169 (33.72) | 320 (37.69) | 1.00 |  |
| rs2736109 | GA | 3156 (49.06) | 1313 (47.71) | 0.94 (0.85-1.04) |  | 3156 (49.06) | 401 (47.23) | 0.88 (0.75-1.03) |  |
|  | AA | 1108 (17.22) | 476 (17.30) | 0.98 (0.86-1.12) |  | 1108 (17.22) | 128 (15.08) | 0.79 (0.63-0.98) |  |
|  | per A allele | |  | 0.98 (0.92-1.05) | 0.562 |  |  | 0.88 (0.80-0.98) | **0.022** |
| **SEARCH** | GG | 3141 (48.88) | 1919 (50.55) | 1.00 |  | 3141 (48.88) | 435 (49.94) | 1.00 |  |
| rs2736108 | GA | 2710 (42.17) | 1581 (41.65) | 0.96 (0.88-1.04) |  | 2710 (42.17) | 369 (42.37) | 0.98 (0.85-1.14) |  |
|  | AA | 575 (8.95) | 296 (7.80) | 0.84 (0.72-0.98) |  | 575 (8.95) | 67 (7.69) | 0.84 (0.64-1.10) |  |
|  | per A allele | |  | 0.93 (0.88-0.99) | **0.031** |  |  | 0.95 (0.85-1.06) | 0.320 |
| ^a^ ABCTB and kConFab cases compared to AOCS controls. ^b^ Adjusted for study in combined analysis. | | | | | | | | | |
